# Palliative radiotherapy for painful non-bone lesions in patients with advanced cancer: a single center retrospective study

**DOI:** 10.1007/s11604-024-01536-0

**Published:** 2024-02-22

**Authors:** Yurika Shindo, Yutaro Koide, Naoya Nagai, Tomoki Kitagawa, Takahiro Aoyama, Hidetoshi Shimizu, Shingo Hashimoto, Hiroyuki Tachibana, Takeshi Kodaira, Shunichi Ishihara, Shinji Naganawa

**Affiliations:** 1 Department of Radiation Oncology, Aichi Cancer Center, Kanokoden 1–1, Chikusa-ku, Nagoya, Aichi 464-0824 Japan; 2 Department of Radiology, Nagoya University Graduate School of Medicine, Nagoya, Japan

**Keywords:** Palliative radiotherapy, Painful metastases, Painful non-bone lesions, Supportive care

## Abstract

**Purpose:**

This retrospective study aimed to assess the efficacy and safety of palliative radiotherapy for painful non-bone lesions in patients with advanced cancer.

**Materials and methods:**

We enrolled patients with painful non-bone lesions who underwent conventional palliative radiotherapy between September 2018 and September 2022. The treatment targets included primary tumor lesions, lymph node metastases, non-bone hematogenous metastases, and other lesions. The primary endpoint was the overall pain response rate in evaluable patients, determined based on the International Consensus Pain Response Endpoint criteria. The secondary endpoints included overall survival, pain recurrence, and adverse events.

**Results:**

Of the 420 screened patients, 142 received palliative radiotherapy for painful non-bone lesions, and 112 were evaluable. A pain response was achieved in 67 patients (60%) of the 112 evaluable patients within a median of 1.2 months. Among these patients, 25 exhibited complete response, 42 partial response, 18 indeterminate response, and 27 pain progression. The median survival time was 5.5 months, recorded at a median follow-up of 6.0 months, during which 67 patients died. Multivariate analysis identified poor performance status scores of 2–4, opioid use, and re-irradiation as independent factors associated with a reduced likelihood of achieving a pain response. Pain recurrence occurred in 18 patients over a median of 4.1 months. Seventeen patients had grade 1–2 adverse events, while none experienced grade 3 or higher toxicity.

**Conclusion:**

Palliative radiotherapy can potentially be a safe and well-tolerated modality for managing painful non-bone lesions, with a low rate of adverse events.

## Introduction

Pain is one of the most common symptoms experienced by patients with cancer, up to 75% of patients experience pain during their illness [1, 2]. Palliative radiotherapy has been used in the management of cancer-related pain, particularly for bone metastases, owing to its rapid and substantial analgesic effects, as evidenced by several systematic reviews and meta-analyses [3–6].

Palliative radiotherapy is used to manage pain from non-bone lesions, such as those in the lymph nodes, skin, soft tissues, and solid organs. However, existing evidence supporting its analgesic efficacy in these non-bone lesions is less substantial, as this evidence is often derived from studies examining individual organ systems [7–14]. The analgesic effect of radiotherapy on all non-bone lesions involving different sites has not been studied in painful bone metastases. In addition, the optimal radiation dose and fractionation schedule for non-bone lesions remains unclear.

This study aimed to evaluate the analgesic efficacy of palliative radiation therapy for painful non-bone lesions based on the International Consensus Pain Response Endpoints (ICPRE) criteria. These criteria have been commonly used in previous studies examining patients with bone metastases, allowing comparison of response rates. The results of this study are expected to provide valuable insights into the optimal use of palliative radiation therapy for the management of pain associated with painful non-bone tumors.

## Materials and methods

### Study design and participants

This retrospective cohort study was conducted on patients with painful non-bone tumors who underwent palliative radiotherapy at our institution between September 2018 and September 2022. This study was approved by our institutional review board, and all participants provided informed consent with an opt-out form that stated that participants were included unless they explicitly decided to exclude themselves. Patients who (1) were diagnosed with painful non-bone lesions, (2) experienced pain related to the targeted tumor, and (3) underwent palliative radiotherapy for pain relief were included. Palliative radiotherapy is defined as radiotherapy aimed at relieving the symptoms related to specific lesions. However, this study did not include patients who underwent palliative radiotherapy for non-bone lesions to relieve symptoms other than pain, such as gastrointestinal stenosis or bleeding. The intended target non-bone lesions for this study were primary tumor lesions, lymph node metastases, hematogenous metastases other than bone metastases, and other lesions (including pleural/peritoneal/meningeal dissemination). Herein, palliative radiotherapy for non-bone lesions was performed at our institution using the three-dimensional conformal radiation method. Therefore, this study did not include data on intensity-modulated radiation therapy, volumetrically modulated arch therapy, or stereotactic body radiation therapy. Patient follow-up for this study ended on May 30, 2023, and those who were alive or lost to follow-up at this time were censored.

**Fig. 1:**
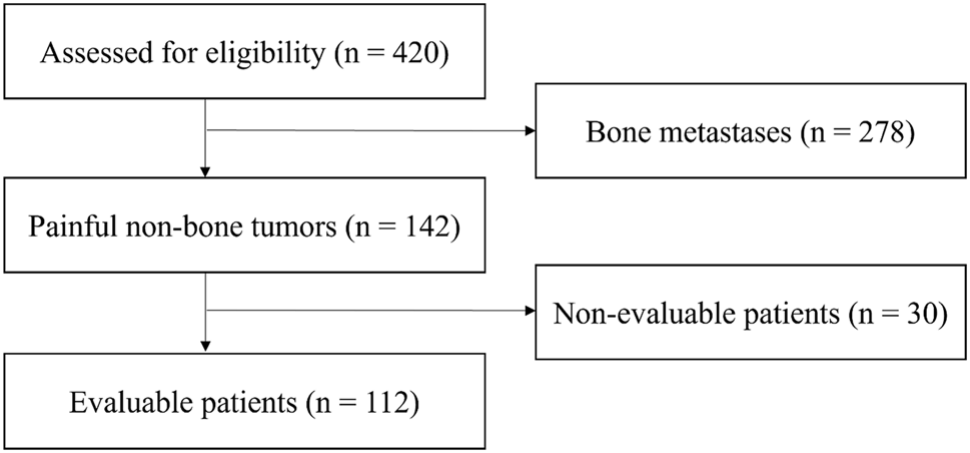
Consort diagram of this study

Figure 1 presents a consort diagram of the study process. We identified 142 patients with painful non-bone lesions among the 420 enrolled patients. Thirty patients were unable to assess their pain response because they were lost to follow-up after radiotherapy. After excluding these 30 non-evaluable patients, 112 evaluable patients were included in the analysis.

### Data collection

We collected data on patient and tumor characteristics, radiation dose and fractionation schedule, worst pain scores, analgesic medication use, imaging findings, adverse events, and survival. Pain scores were determined based on the patient’s self-reported worst pain experienced in the past 3 days, rated using the Numeric Rating Scale (NRS); the scores ranged from 0 to 10, with higher scores indicating more severe pain [15]. Patients with an NRS score of 0 were excluded. Analgesic medication use was recorded as the daily oral morphine equivalent dose (OMED) calculated based on the National Comprehensive Cancer Network guidelines [16].

The pain response was assessed using the ICPRE criteria [17]. Overall response (OR) included complete response (CR) and partial response (PR). CR was defined as a pain score of 0 at the treated site with no concomitant increase in analgesic intake. PR was defined as a pain reduction of 2 or more without an analgesic increase or an analgesic reduction of 25% or more from baseline without an increase in the severity of pain. Pain progression (PP) was defined as an increase in pain score of 2 or more above the baseline with stable OMED or an increase of 25% or more in OMED compared with the baseline with a stable pain score or a pain score of 1 point above the baseline. Indeterminate response (IR) was defined as a response other than CR, PR, or PP.

### Endpoint

The primary endpoint was the pain response rate in evaluable patients, defined as the proportion of the overall response based on pain scores and analgesic medication use within 4 months of radiotherapy. The secondary endpoints were adverse events, survival rates, and pain recurrence. Pain recurrence was defined as PP occurring after a pain response, but the baseline pain score for determining PP was changed to the minimum score after the pain response.

### Statistical analysis

All the explanatory variables were treated as categorical variables: Eastern Cooperative Oncology Group Performance Status (0–1 vs. 2–4), NRS (2–6 vs. 7–10), age (< 66 vs. ≥ 66), sex (female vs. male), OMED (< 60 mg vs. ≥ 60 mg), radiation dose (≥ 40 Gy_10_ BED_10_ vs. < 40 Gy_10_ BED_10_), and the history of radiotherapy (yes vs. no). In addition, the target lesions were classified as primary tumor lesions, lymph node metastases, non-bone hematogenous metastases, or others. To explore the factors affecting the pain response, univariate and multivariate logistic regression analyses were performed using the above covariates as explanatory variables. Variables with a *P*-value < 0.05 after a univariate analysis were selected for inclusion in the multivariate model.

Survival curves were estimated using the Kaplan–Meier method, and the differences between groups were compared using the log-rank test. Statistical significance was set at a *P*-value of < 0.05. All statistical analyses were performed using the R statistical software version 4.2.2 (The R Foundation for Statistical Computing, Vienna, Austria).

## Results

### Patient

The patient characteristics are shown in Table 1. The most common target lesions were primary lesions (28%) and lymph node metastases (28%). The doses and fractions (fx) commonly used were 30 Gy/10 fx (25%), 24 Gy/6 fx (25%), 20 Gy/5 fx (22%), and 8 Gy/1 fx (7%). Twenty-three patients (21%) had a history of irradiation for the same lesion (i.e., re-irradiation cases).

**Table 1:** Patient characteristics

Factor	Group	Overall
Sample size, no.		112
Age	Median (range)	66 (31–85)
Sex, (%)	Female	50 (44.6)
	Male	62 (55.4)
Primary tumor, (%)	GI	34 (30.4)
	Lung	18 (16.1)
	Breast	11 (9.8)
	Sarcoma	18 (16.1)
	Other	31 (27.7)
Target lesion, (%)	Primary lesions	31 (27.7)
	Lymph nodes metastases	31 (27.7)
	Hematologic metastases	20 (17.9)
	Other lesions	30 (26.8)
ECOG performance status, (%)	0–1	80 (71.4)
	2	25 (22.3)
	3	6 (5.4)
	4	1 (0.9)
NRS, (%)	2–6	67 (59.8)
	7–10	45 (40.2)
OMED, (%)	No opioids (0/NSAIDs only)	39 (34.8)
	< 60 mg/day	38 (33.9)
	≥ 60 mg/day	35 (31.2)
Radiation dose, Gy_10_ in BED10, (%)	Median (range), Gy_10_	33.6 (4–72)
	< 15 Gy_10_ (e.g., 8 Gy/1 fx)	10 (8.9)
	15–40 Gy_10_ (e.g., 20 Gy/5 fx, 30 Gy/10 fx)	81 (72.3)
	> 40 Gy_10_	21 (18.8)
History of radiotherapy, (%)	No	89 (79.5)
	Yes	23 (20.5)

*GI* gastrointestinal tumor; *ECOG* eastern cooperative oncology group; *NRS* numerical rating scale; *OMED* oral morphine equivalent dose; *NSAIDs* non-steroidal anti-inflammatory drugs; *BED10* biological effective dose with theα/β ratio of 10; *fx* fractions

### Pain response, survival, and adverse events

At the end of the follow-up, 45 patients were alive, and 67 died. The median follow-up time for the surviving patients was 6.0 months. In 112 evaluable patients, pain response was observed in 67 patients (60%). The median time to evaluation was 1.2 months (interquartile range: 0.83–2.1 months), and 25 (22%), 42 (38%), 18 (16%), and 27 (24%) patients achieved CR, PR, IR, and PP, respectively. During the follow-up period, pain recurrence occurred within a median of 4.1 months (range: 0.83–15.6 months) in 18 of 67 (27%) once-responded patients after radiation therapy. Univariate and multivariate analyses identified the factors associated with pain response (Table 2). Patients with a PS score of 2–4, who received an OMED of ≥ 60 mg/day, and who underwent re-irradiation were significantly less likely to pain respond (odds ratios (95% confidence intervals (CIs)): 0.64 (0.41–0.99), 0.41 (0.23–0.72), and 0.45 (0.28–0.74), respectively (*P*-values: 0.047, 0.0018, and 0.011). The median survival time was 5.5 months (95% CI: 4.5–8.9 months), and the overall survival at 1 year was 36% (95% CI: 25–47%).

**Table 2:** Univariate and multivariate analysis of pain response

Variable (n = 112)	Univariate analysis			Multivariate analysis		
	OR	95% CI	*P*	OR	95% CI	*P*
*ECOG performance status*						
2–4 vs. 0–1	0.46	0.31–0.69	< 0.001*	0.64	0.41–0.99	0.047*
*NRS*						
2–6 vs. 7–10	0.97	0.66–1.43	0.89			
*Age*						
< 66 vs. ≥ 66	0.86	0.58–1.26	0.44			
*Sex*						
Male vs. female	1.48	1.00–2.18	0.049*	1.5	0.99–2.25	0.053
*OMED*						
< 60 mg/day vs. no opioids	0.76	0.47–1.21	0.24	0.83	0.51–1.36	0.46
≥ 60 mg/day vs. no opioids	0.33	0.19–0.56	< 0.001*	0.41	0.23–0.72	0.0018*
*Radiation dose, Gy*_*10*_* (BED10)*						
≥ 40 vs. < 40	0.95	0.51–1.76	0.87			
*History of radiotherapy*						
Yes vs. No	0.45	0.28–0.74	0.0015*	0.51	0.31–0.85	0.011*

*OR* odds ratio; *CI* confidence interval; *ECOG* Eastern cooperative oncology group; *NRS* numerical rating scale; *OMED* oral morphine equivalent dose

*Statistically significant (*P* < 0.05)

Seventeen patients developed grade 1–2 adverse events (15%), including nausea (three), dermatitis (five), mucositis (five), diarrhea (one), headache (one), edema (one), and peripheral neuropathy (one). None of the patients experienced adverse events of grade 3 or higher.

## Discussion

This study evaluated the analgesic effects of palliative radiotherapy on painful non-bone lesions in patients with advanced cancer. The strength of our study is the use of ICPRE to evaluate pain response, which allows a comparison of the response rates between the present study and previous studies on bone metastases. Our study observed a pain response rate of 60%, consistent with previous studies examining the efficacy of palliative radiotherapy for painful bone metastases [3–6]. The observed adverse events were minor, with 15% of patients experiencing grade 1–2 severity. A recent systematic review and meta-analysis by Imano et al. on conventional palliative radiotherapy for painful bone metastases reported an overall response rate of 60% in evaluable patients [3]. Saito et al. reported overall response rate of 51–54% in evaluable patients in their observational study on palliative radiotherapy for painful non-bone lesions [7]. Table 3 summarizes the previous results of palliative radiotherapy for painful non-bone lesions [7–9, 12–14]. Although limited data are available for non-bone lesions, pain response rates were approximately 60% in previous studies, similar to the reports of previous studies on painful bone metastases. Our study had a relatively large sample size. These findings indicate a potential similarity in the analgesic effects of palliative radiotherapy for both bone metastases and various non-bone lesions. However, the current evidence is limited, highlighting the need for further studies on palliative radiotherapy for painful non-bone lesions.

**Table 3:** Results of conventional palliative radiotherapy for non-bone painful lesions

Author (Reference)	N	Target sites	Dose, Gy	Pain response rate, %	Use of ICPRE
Current study	112	Lymph nodes/Skin/Soft tissue/Solid organs	8–60	59.8	Yes
Saito [7]	63	Primary tumor/Lymph nodes/Hematogenous/Pleural dissemination/Other lesions	6–60	51–54	Yes
Yamaguchi [8]	21	Lymph nodes	8–60	57–67	Yes
Erridge [9]	53	Chest	10–30	50–84	No
Yeung [12]	31	Liver	8	55	No
Ito [13]	28	Liver	8	64	Yes
Dawson [14]	42	Liver	8	67	No

*ICPRE* international consensus pain response endpoints

The radiation doses used in this study were generally consistent with the standard palliative radiation therapy doses used for painful bone metastasis. Most patients received 20–30 Gy doses in 5–10 divided doses and only 7% of patients underwent 8 Gy in a single fx. In previous studies about radiotherapy for painful bone metastases, a single 8 Gy dose provided analgesia that is comparable to that achieved using longer schedules [3, 4, 18–20]. The efficacy of a single 8 Gy irradiation for painful non-bone lesions is not as well established as that for painful bone metastases. Although the present study did not allow comparison by target sites because of a small sample size, considering the effectiveness of a single 8 Gy irradiation for painful liver tumors in previous studies, a single 8 Gy irradiation for non-bone lesions may be effective and not specific to the target sites [12–14]. Our cohort included poor prognosis patients with a median survival time of 5.5 months, which suggests that excessively long irradiation schedules for pain management should be avoided. Future trials are warranted to determine the optimal radiation dose and fractions for patients with painful non-bone lesions.

The present study has several limitations. First, this was a retrospective study with a relatively small sample size, and the timing of the response assessment was not uniform, which may limit the generalizability of our findings. Second, the follow-up period was relatively short, which may have led to the underestimation of pain recurrence and adverse events. Third, the radiation dose and fractionation schedule varied among the patients, which may have influenced the response to radiotherapy. Further studies are needed to evaluate the optimal radiation dose and fractionation for palliative radiotherapy of painful non-bone lesions.

In conclusion, palliative radiotherapy is a safe and well-tolerated modality for managing painful non-bone lesions, with a low rate of adverse events.
